# An Interoperable Communication Framework for Grid Frequency Regulation Support from Microgrids

**DOI:** 10.3390/s21134555

**Published:** 2021-07-02

**Authors:** Lilia Tightiz, Hyosik Yang, Hassan Bevrani

**Affiliations:** 1 Department of Computer Engineering, Sejong University, 209, Neungdong-ro, Gwangjin-gu, Seoul 05006, Korea; liliatightiz@sju.ac.kr; 2 Department of Electrical and Computer Engineering, University of Kurdistan, Sanandaj 66177-15175, Iran; bevrani@uok.ac.ir

**Keywords:** microgrid, frequency regulation, IEC 61850, data distribution services, variable of interests

## Abstract

Renewable energy sources, which are controllable under the management of the microgrids with the contribution of energy storage systems and smart inverters, can support power system frequency regulation along with traditionally frequency control providers. This issue will not be viable without a robust communication architecture that meets all communication specification requirements of frequency regulation, including latency, reliability, and security. Therefore, this paper focuses on providing a communication framework of interacting between the power grid management system and microgrid central controller. In this scenario, the microgrid control center is integrated into the utility grid as a frequency regulation supporter for the main grid. This communication structure emulates the information model of the IEC 61850 protocol to meet interoperability. By employing IoT’s transmission protocol data distribution services, the structure satisfies the communication requirements for interacting in the wide-area network. This paper represents an interoperable information model for the microgrid central controller and power system management sectors’ interactions based on the IEC 61850–8–2 standard. Furthermore, we evaluate our scenario by measuring the latency, reliability, and security performance of data distribution services on a real communication testbed.

## 1. Introduction

Microgrids (MG) are known as the main blocks of a smart grid [1]. Based on the IEEE Std 2030.7-2017 MG is defined as “A group of interconnected loads and distributed energy resources with clearly defined electrical boundaries that acts as a single controllable entity with respect to the grid and can connect and disconnect from the grid to enable it to operate in both grid-connected or island modes” [2]. MG penetration has increased due to the reduction in the cost of electricity transmission infrastructure from utilizing distributed energy resources (DER) [3,4]. Employing renewable energy sources (RES) in MG due to their uncountable profits will dominate other kinds of DER. However, the nature of RES is intermittent. Therefore, the primary role of inverters, i.e., power conversion and energy feeding, has been developed to provide dispatchable sources, able to overcome RES randomness as well as meet tied power system frequency and voltage deviations. Smart inverters offer services such as harmonics, voltage, and frequency control to achieve cooperation between RES and the utility grid [5,6,7,8,9]. Along with smart inverters, the presence of energy storage systems (ESS) and responsive loads (RL) allows the MGs to participate in the electricity market as a prosumer, selling overproduced electricity and buying in case of resources unavailability or system failure in grid-connected mode [10]. These characteristics have brought the opportunity of employing MG as an ancillary service (AS) provider of utility grid, including voltage control, black-start aid, and especially frequency regulation (FR) support [11,12,13,14]. This scheme, in the 3DMicroGrid project, arranges MG set points to assist transmission system operator (TSO) in frequency control and distribution system operator (DSO) in voltage control and load curtailment [15]. Additionally, in Belgium, TSO maintains tertiary FR through the capacity reserve, namely R3 dynamic profile and R3 aggregated power plant by deploying RES, ESS, and RL instead of conventional bulk generation units [16]. However, these cases would not be applicable without a robust, real-time, reliable, and secure communication architecture happening in the wide-area network (WAN) because the provision of AS and particularly FR in the power grid should be available in a limited time. With the advent of smart grid, the flow of power and data in the power system alternated to a bidirectional style. Deploying IoT technology facilitates communication based on the Internet infrastructure in WAN. However, transmission protocols play a crucial role in fulfilling a precondition of smart grid penetration increment: to provide robust, real-time, reliable, and secure communication [17]. The IEC 61850 standard series, which were initially communication protocol of automation for power system’s substations, have been developed to make whole horizons of power system intelligent. IEC 61850–7–420 and IEC 61850–90–7 specify information model for MG interactions and introduce use cases about how to exchange information among the DERs and supervisory of the power system [18,19]. Moreover, the recently published part of IEC 61850, part 8–2, used extensible markup language (XML) parsing to determine how to map communication services onto IoT protocols, facilitating interaction in WAN based on the Internet environment [20]. In response to communication requirements of interaction in smart grid, mapping the IEC 61850 onto data distribution services (DDS) will be considered in this paper. The DDS is a data-centric middleware surviving bottleneck broker drawback of other IoT protocols, provides interoperability, and supplies robust and reliable communication with a wide range of quality of services (QoS) for communication in WAN.

Scholars studied the utilization of the microgrid as a service provider of the main grid in two different domains, including planning optimization algorithm and communication structure according to the Table 1 [12,13,21,22,23,24,25,26,27,28,29,30,31,32,33]. Since for the first time in this paper we focus on preparing interoperable schema for micro-grid as the utility grid AS provider with IoT protocols assistant, we proposed this research area’s related studies in detail here. Youssef et al. [25] investigated a real-time energy management system’s (EMS) communication requirements of MG and applied the DDS as middleware to implement this communication architecture, exchanging data over the Ethernet network. Voltage regulation of active distribution network (ADN) was established by the usage of DER and ESS equipped with smart inverters in [27]. Additionally, authors in this paper considered the elements of ADN as a multi-agent system (MAS) and utilized DDS protocol to provide fast and reliable communication. Esfahani et al. [26] derived the benefits of real-time communication by deploying DDS in a multi-MG environment energy market with the presence of distributed ESS, DER, and RL, minimizing the dependency of MG to utility grid. Reference [28] applied DDS for communication inside MG to coordinate inverters and control the voltage and frequency of MG during islanded detection and grid-connected mode. However, by not proposing a data model based on the IEC 61850 standard series, there is a lack of interoperability, which is the main drawback of the above-mentioned works.

Habib et al. [29,30] provided a fast protection scheme for MG by applying DDS gateway on transmission lines as middleware to satisfy the time-constrained requirements of IEC 61850’s critical messages, i.e., generic object oriented substation event (GOOSE) and sampled measured values messages (SMV) in a local area network (LAN). Frequency control of MG as an interactive element of utility grid implemented in [31] by preparing an information model based on the IEC 61850 mapped to the DDS. This paper implemented agent communication language (ACL) for communication among utility and agents instead of XML, which is a message format of the IEC 61850.

**Table 1:** Microgrid as ancillary service provision, related works’ objectives comparison.

Ref	Study Area		Main Objective	Communication Structure						
	Planning Optimization Algorithm	Design Communication Structure		MG Interaction with Main Grid	Communication Network	Interoperability		Implementation Platform	Time Constraint Investigation	Communication Security Investigation
						IEC 61850 Information Model	IEC 61850 Message Format			
[31] 2016	YES	YES	Multi-agent based MG control system	YES	LAN	Referred but not proposed	NO	Real LAN testbed	YES	NO
[25] 2017	YES	YES	Real-time microgrid EMS	YES	LAN	NO	NO	Real LAN testbed	NO	NO
[27] 2018	YES	YES	Voltage regulation in active distribution network	YES	LAN	NO	NO	Real LAN testbed	YES	NO
[26] 2019	YES	YES	Multiagent market for multi-MG system	YES	LAN	NO	NO	Real LAN testbed	NO	NO
[28] 2019	NO	YES	Communication structure for control voltage and frequency of MG	NO	LAN	NO	NO	Real LAN testbed	NO	NO
[29] 2019	YES	YES	MG protection	NO	LAN	Referred but not proposed	IEC 61850-1	Real LAN testbed	YES	NO
[12] 2020	YES	NO	MG stability provision to act as ancillary service provider of main grid	YES	NO	NO	NO	NO	NO	NO
[13] 2020	YES	NO	MG as active distribution network offering frequency regulation service provider of main grid	YES	NO	NO	NO	NO	NO	NO
[30] 2020	NO	YES	MG protection	NO	LAN	Referred but not proposed	IEC 61850-1	Real LAN testbed	YES	NO
[34] 2021	YES	NO	MG as a participant of power grid protection schema	YES	NO	NO	NO	NO	NO	NO
[32] 2021	NO	YES	communication structure for LFC in an islanded MG	NO	WAN (simulated environment)	YES	IEC 61850-1 (LAN)	Real LAN testbed and simulated WAN	YES	NO
[33] 2021	YES	YES	communication structure for a fault detection and system restoration in the Multi-MG system	YES	LAN	YES	IEC 61850-1	Ethernet based HIL	YES	NO
Present Work	YES	YES	Interoperable communication structure for MG as main grid ancillary service provider	YES	WAN (main grid) LAN (inside MG)	YES	IEC 61850-1 (LAN) IEC 61850-2 (WAN)	Real WAN/LAN testbed	YES	YES

The before-mentioned practices applied the DDS with a dedicated communication network infrastructure for MG internal interactions. Moreover, their platforms were mostly implemented in the LAN environment. In a recent effort, Aftab et al. [32] planned an information model based on IEC 61850 for an islanded MG based on IEC 61850 and examined load frequency control (LFC) inside the islanded MG. The authors in this paper arranged WAN on a simulator environment and evaluated latency for their LFC scenarios. In the same line of thought, Hong et al. [33] applied IEC 61850 to provide a fault detection and system restoration in the Multi-MG system with the assistance of hardware-in-loop (HIL) testbed. The authors deployed an Ethernet-based infrastructure for communication which represents a LAN arrangement. However, to prove the implementation possibility of an interoperable, fast, cost-effective, reliable, and secure interaction for MG in the real WAN environment, we utilized DDS as a robust IoT protocol to transport IEC 61850 messages format for the scenario of MG as the utility grid FR support on the Internet. Hence, the contribution of this paper will be:

- Define information model of MG elements and service requirements based on IEC 61850 standard in the utility grid FR-support scenario.
- Provide experimental setup of communication infrastructure based on the DDS protocol in the WAN environment.
- Investigate results according to the FR communication requirements fulfillment.

The remainder of this paper is organized as follows: Section 2 provides the MG structure and control methods to act as an FR-support of utility grid and presents its set of variables of interest. Section 3 investigates the communication model and services of our scenario based on IEC 61850 and maps onto the DDS protocol to obtain communication in the Internet infrastructure. Section 4 is devoted to the experimental setup and its results to prove the effectiveness of our proposed model. Ultimately, this paper ceases in Section 5 as a conclusion.

## 2. Scenario Arrangement for MG Participation in Power System FR

### 2.1. Power System FR with the MG Presence

Managing reliability and stability of the power system make it necessary to keep frequency in a defined boundary. Equilibrium between active power generation and consumption provides FR. Frequency control in the power grid has been divided into three levels: primary, secondary, and tertiary control. Primary control is automatic and responded to in seconds. The elements cooperating in this level of frequency control are synchronous generators (SG). Traditionally, governors in SG apply the droop control method to accommodate primary control. Furthermore, large scale RES power plants (LRES) and ESS (LESS) equipped with smart inverters as well as responsive loads (RL) through demand response scheduling can take part in this fast required response control level. Secondary control that is called load frequency control (LFC), which is automatic and centralized as the main function of automatic generation control (AGC), should be responded to in a few seconds to minutes. Secondary control, implemented by the control loop, compensates deviations of frequency beyond the coverage of primary control level and determines the participation rate of each generation unit in this process. Active power tie-line deviation is added as a tertiary control level to the secondary level since the power grid is an integrated multi-area. The tertiary level is manual and should be controlled in minutes. Active power deviation in power system with high penetration of MG, LRES, and LESS can be calculated as follows [35,36].

(1) $$\begin{array}{c} \Delta P_{SG}\pm \Delta P_{LESS}+\Delta P_{LRES}+\Delta P_{RL}\pm \Delta P_{MG}-\Delta P_{d}\\ =\;2H\frac{d\Delta f(t)}{dt}+D\Delta f(t) \end{array}$$

where;

$\Delta P_{SG}$: deviation in power generated by SG;
$\Delta P_{LESS}$: deviation in power delivered or consumed by large scale ESS;
$\Delta P_{LRES}$: deviation in power generated by large sclae RES;
$\Delta P_{RL}$: deviation in the power demanded by RL;
$\Delta P_{MG}$: deviation in power delivered or consumed by MG;
$\Delta P_{d}$: disturbances in loads power demand;
*H*: system inertia provided by synchronous generators;
*D*: power system damping coefficient.

MG in our proposal will be a collection of micro-SG (mSG), small scale ESS, and RES equipped with smart inverters, noncritical loads can participate in demand response known as RL, and critical loads, which should be served continuously. Therefore, MG’s output power, which can be positive in generative mode of MG and negative in consumer mode, w.r.t. its elements states is calculated as shown in (2).

(2) $$\begin{array}{c} \Delta P_{RL}+\Delta P_{RES}+\Delta P_{mSG}\pm \Delta P_{ESS}-\Delta P_{d}\\ =\pm \Delta P_{MG} \end{array}$$

where;

$\Delta P_{RES}$: deviation in power generated by RES in MG domain;
$\Delta P_{mSG}$: deviation in power generated by microsynchronous generators in MG domain;
$\Delta P_{ESS}$: deviation in power delivered or consumed by ESS in MG domain;
$\Delta P_{RL}$: deviation in the power demanded by RL in MG domain;
$\Delta P_{d}$: disturbances in loads power demand in MG domain.

**Figure 1:**
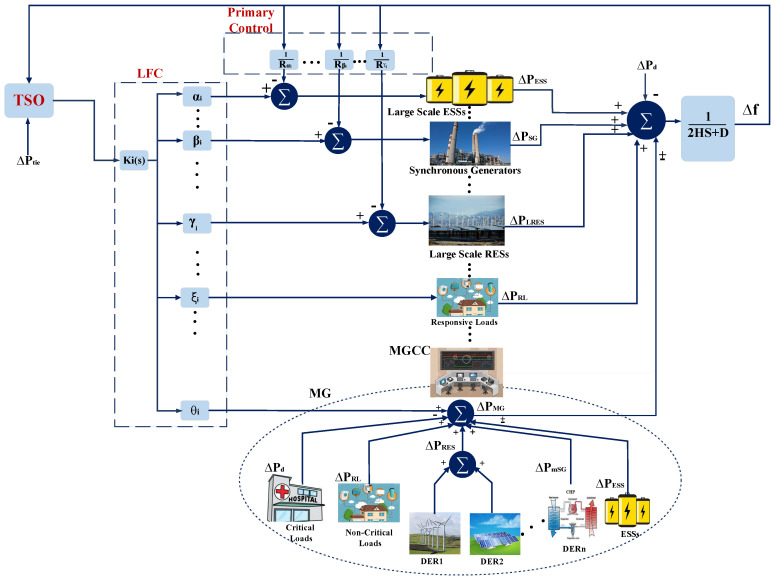
Frequency control in the power system with the MG presence. The power system includes RES and SG-based power plants, Large scale ESSs, RLs, and MGs. Each element supports FR with the portion that is specified by TSO. The MG also includes RES and SG-based power plants, ESSs, critical, and noncritical loads that contribute to the FR in the MG domain. The MG elements are under the supervisory of MGCC.

Figure 1 visualizes the basic frequency control levels in the power system with the MG presence. The TSO monitors the frequency of grid and determines the share of each generation unit in the FR. *R* is the droop control characteristic of each generation unit. $K_{i}\left(s\right)$, which is the transfer function of the secondary control level for each participant, with assistance from $\alpha_{i}$, $\beta_{i}$, $\gamma_{i}$, $\xi_{i}$, and $\theta_{i}$ coefficients show the participation factor of each unit in frequency control. Regarding this definition, we can estimate each generation unit portion shown below:

(3) $$\Delta P_{ESS}=\alpha i.K_{i}\left(s\right)$$

(4) $$\Delta P_{SG}=\beta i.K_{i}\left(s\right)$$

(5) $$\Delta P_{LRES}=\gamma i.K_{i}\left(s\right)$$

(6) $$\Delta P_{RL}=\xi i.K_{i}\left(s\right)$$

(7) $$\Delta P_{MG}=\theta i.K_{i}\left(s\right)$$

(8) αi+βi+γi+ξi+θi=1

(9) i={0,1,...,n};

where;

*i*: number of actors in the FR;
α: participation factor of ESS in FR;
β: participation factor of SG in FR;
γ: participation factor of LRES in FR;
ξ: participation factor of RL in FR.
θ: participation factor of MG in FR.

Equations (3) to (7) describes the amount of active power that should be delivered or consumed by ESS, SG, LRES, RL, and MG, respectively to contribute to LFC while the number of participants in this schema is according to Equation (9). Equation (8) shows the necessity of equilibrium in power consumption and generation in the power system. Table 2 shows time constraints in frequency control based on the time of processing $T_{p}$ and resources participation detection $T_{d}$ according to the European network of transmission system operators (ENTSO) regulations [37].

### 2.2. MG Structure and Control Methodologies

**Figure 2:**
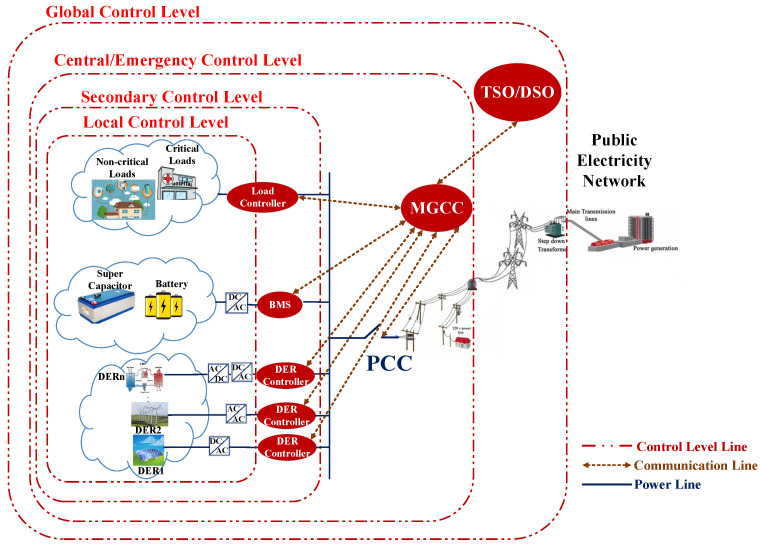
Four different levels of control in the MG. The MG under the central control unit called MGCC interconnects the utility grid.

According to the initial objectives of MG advent in version 2003 of the IEEE 1547 standard, the MG works in grid-connected mode and supplies consumers by the grid. However, in the case of any failure, which could result in deviation of frequency and the voltage of the main grid, MG should disconnect from the grid and supply whole or part of loads autonomously by the ESS, which has been charged by RES during the grid-connected mode [38]. In the 2018 version of IEEE 1547 standard, this strategy improved by adding voltage and frequency ride-through capabilities to the RES interfaces in abnormal situations during the grid-connected mode [39,40]. These characteristics will be served by smart inverters. In addition to their typical responsibility of converting resources output power to grid desired level, smart inverters control voltage and frequency of sources while adjusting to grid set points. Applying this enhancement in the MG control methodology accelerates the pace of MG integration to the utility grid as a service provider. As can be seen in Figure 2, MG control methodology contains four levels: local, secondary, central/emergency, and global control levels [41]. While local control level ensures autonomous stability of each MG’s power source through voltage and current internal loops, the secondary control level using communication adjusts power sources voltage and frequency in response to any fluctuation in loads or power sources. Smart inverters serve local and secondary control levels of MG. Central/emergency control level determines islanded or grid-connected mode of MG by implementing EMS and protection schemas in both normal situation and contingencies. The MG central controller (MGCC) represents central/emergency control level of MG. The MGCC interacts with a higher level of power system, where global control level is located. Global control level, which can be considered as DMS/DSO or power distribution system’s EMS, is responsible for coordinating the performance of MG with neighbor MGs and the utility grid. To contribute in FR support of the main grid scenario, MG should provide stable frequency and voltage and a specified amount of output power according to the grid requirements. This statement means each MGCC receives set points from the DSO to adjust its actions in the grid-connected mode [14]. The IEC 61850-–90-–7 provides the communication framework for MG, which interacts with its supervisory level and considers smart inverters deployment for the RES to manage their intermittency and local and secondary control level implementation. Furthermore, the IEC 61850-–90-–7 determine predefined control modes for the DER and ESS. These modes can be addressed as the central level control. These schedules will result in a reduction in communication bandwidth requirements. In Algorithm 1, we determine which variables of interest to communicate with for the FR support of the utility grid by the MG. Additionally, since MG can import or export power to the main grid at the PCC, MG can communicate with DSO and participate in the FR provision for the main grid through the MGCC based on Algorithm 1. To control frequency deviation, TSO first sends a request for active power adjustment to regulate frequency and determines set points for all parties that can assist FR. Furthermore, DSO calculates the desired power level of each available unit according to their predefined cost functions and requests those set points. The MGCC receives commands, estimates which predefined schedule is suitable for DER, and issues related commands. This estimation is taken from information received by DSO and MGCC through reports sent by DER according to DS93 function of IEC 61850–-90-–7. MGCC by analyzing received information issues different functions of IEC 61850 to DER, ESS, and RL including: connecting to or disconnecting from the grid (Function INV1), changing their desired level of active power generation (INV2), and utilizing ESS (INV4).

**Algorithm 1:** Interoperable algorithm for contribution of the MG in the utility grid FR based on IEC 61850.

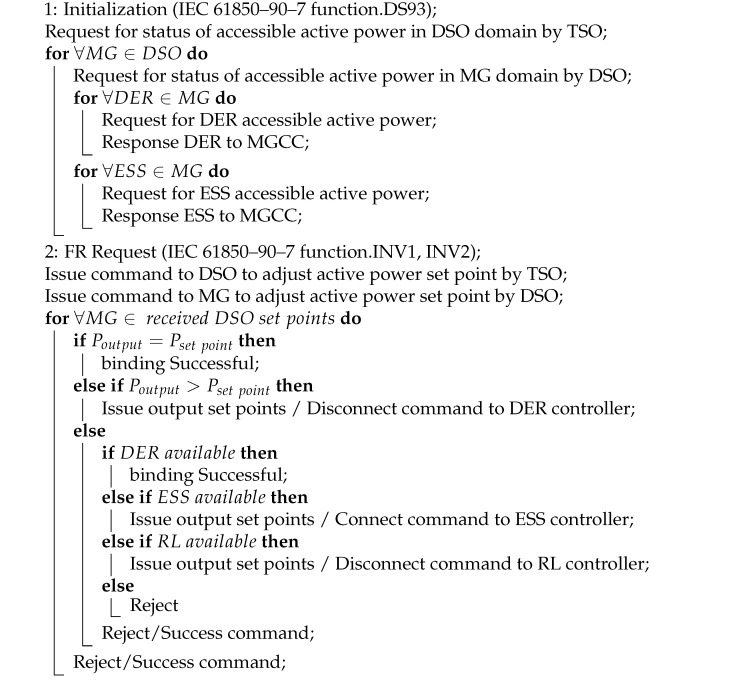



## 3. The Proposed Communication Framework

### 3.1. Information Model Based on IEC 61850

**Figure 3:**
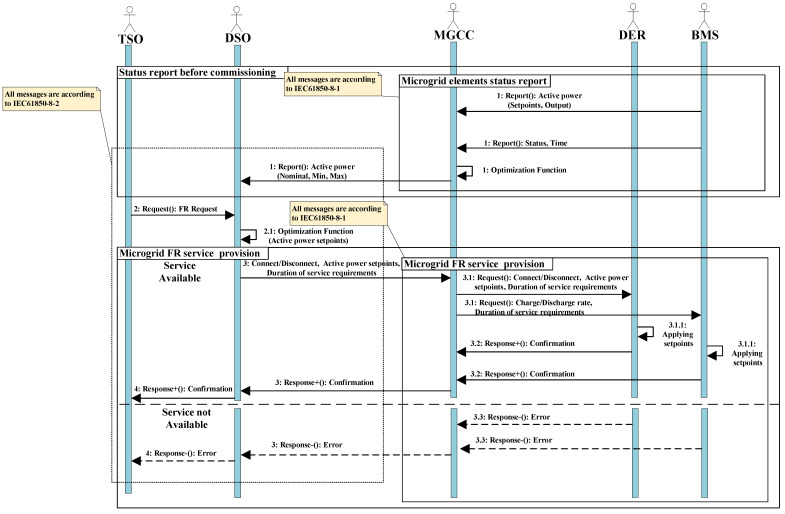
Sequence diagram of FR support from MG. The message format for interaction inside microgrid, which is LAN, is according to the IEC 61850–8–1 and IEC 61850–8–2 is used for communication in WAN, between the MGCC and the utility grid supervisory unit i.e., DSO/TSO.

IEC 61850 is a communication standard that provides an infrastructure for information exchange in the power grid by definition of information model. In addition to this benefit, the IEC 61850 also supplies an assortment of services for interaction of model elements and mapping services to the specific communication protocol [42]. Therefore, the configuration of robust communication, which assures MG as FR supporter, requires a comprehensive information model based on predefined control methodology of grid which is introduced in Section 2 and displayed in Figure 1. Physical devices, called intelligent electronic devices (IED), include the functions in IEC 61850. Furthermore, logical nodes demonstrate each function of IED and include several data objects, which delineate information interests of each IED’s logical nodes. Therefore, in this paper, we consider battery management system (BMS) and DER controllers as IED and servers of IEC 61850 interacting with the MGCC. Since this interaction happens in LAN, they use manufacturing message specification (MMS) protocol and all messages are according to IEC 61850–8–1. In this communication framework, we assume that MGCC has its own logical nodes and interacts with DSO over the WAN. The MGCC and DSO apply IEC 61850–8–2 message format and DDS protocol for communication through the Internet in a WAN environment. Since the implementation of the scenario utilizes the functions in part 90–7 of the standard, logical nodes of those functions should be determined. Figure 3 shows a sequence diagram of the proposed scenario and Table 3 depicts logical nodes of this diagram actors with the sequences of interactions and service requirements. Table 2 also delineates the required set points for each step of communication.

### 3.2. Service Mapping Requirements Based on DDS

The information model and service requirements of our FR scenario needs an infrastructure that ensures real-time, reliable, and secure communication in the WAN. The DDS middleware, standardized by the object management group (OMG), provides these characteristics by the publish–subscribe message pattern. The DDS derives a benefit that participants do not require to know each other from applying discovery methods, including data-centric publisher–subscriber or real-time publisher–subscriber. Therefore, the DDS is a brokerless information exchange protocol without the risk of bottleneck failure. Data-centric publisher–subscriber includes the domain in which all communication entities are placed and interact. Domain participants, including Data Readers, Data Writers, Publishers, and Subscribers gain access to data, based on domain topic and type. As shown in Table 3, the implementation of the FR scenario requires the mapping IEC 61850 services on the DDS protocol. In our scenario, topics are the requests, responses, and reports of IEC 61850 services. Each Data Writer and Data Reader is dedicated to a special topic and is responsible for the marshaling and demarshaling of information. To satisfy interoperability, information exchange based on IEC 61850–8–2 message format utilizes XML parser for marshaling and demarshaling information. Publisher and Subscriber can interact with Data Writer and Reader respectively, corresponding with several topics. The DDS offers 22 levels of QoS that guarantees the quality of each domain entity interaction, even with UDP as a transport layer. It also supports TCP transport layer [43]. In step one, each entity of our proposal reports its condition to the supervisory levels. This information is time-dependent and should be updated; therefore, QoS should set Deadline and History for Data Writers and Data Readers. Other steps have a request–response pattern, requiring reliability in information exchange, then QoS of Data Writer and Reader of those steps set to Reliable mode. Interaction in WAN is on the Internet to avoid the necessity of providing the dedicated communication infrastructure. Therefore, it is vital to make this communication secure against illegitimate publications, disguised subscriptions, data theft, replay attack, and unauthorized access. To address this problem in this paper, we applied three service plugin interfaces, including authentication, access control, and cryptographic implemented by the DDS security model [44].

## 4. Experimental Investigations

### 4.1. Experimental Setup

**Figure 4:**
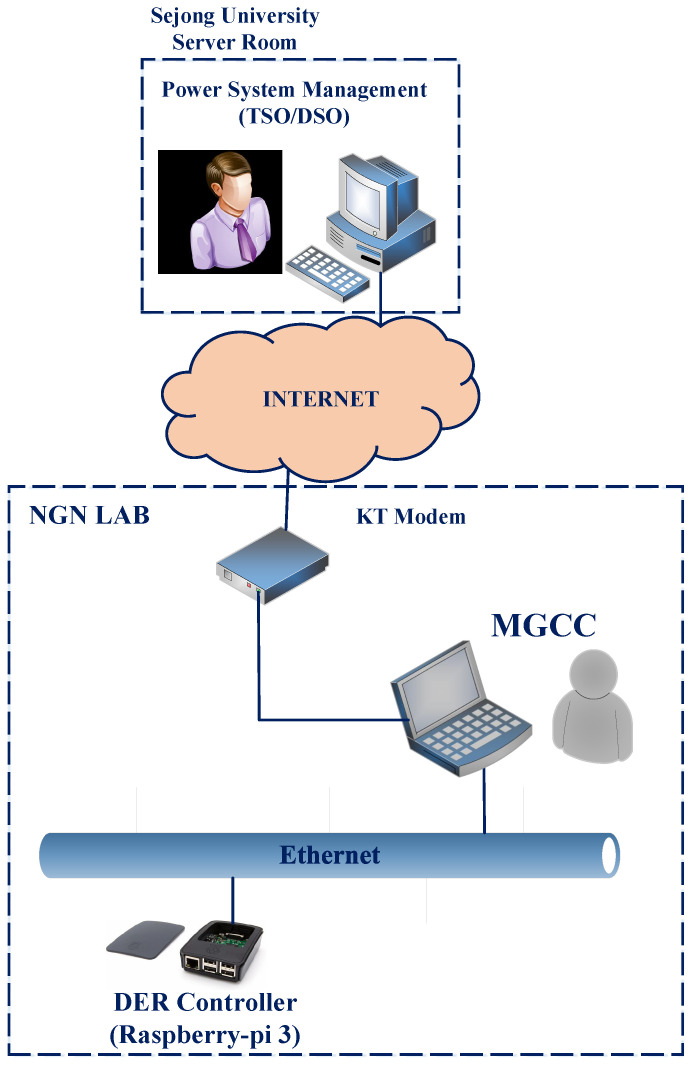
MG as FR-support network infrastructure (Experimental setup). The MG is arranged in the NGN Lab’s LAN, where DER controllers and MGCC are located. The WAN for interaction between MGCC and TSO/DSO is implemented through the Internet connection between NGN Lab and the Sejong University server room.

Figure 4 provides the experimental setup for the evaluation of the time constraint specification requirements of FR. As can be seen in this figure, LAN, the communication structure inside MG, is implemented in the LAN of the next-generation network (NGN) laboratory in Sejong university. Additionally, WAN, the Internet connection between the NGN laboratory and the Sejong University server room, accommodated by the Korean Telecommunication (KT) line. To develop the server and client of IEC 61850, we use MMS-Lite API which is an IEC 61850 data model and protocol stack implementation by systems integration specialists company (SISCO) with C language. The server of the IEC 61850 is a raspberry-pi and considered as the DER controller that connects to the client of the IEC 61850 which is MGCC. The DDS server acts on the laptop computer, which the MGCC is implemented on. Table 4 depicts our experimental network equipment and their software and hardware infrastructures in detail. It is noted that in the experimental setup arrangement we postpone TSO interaction with DSO, since it has the same arrangement of interaction between DSO and MGCC based on DDS protocol. To apply its effect on the latency of interaction in our scenario, we added one step request and reply between DSO and MGCC in our case studies. Additionally, the number of hops between DSO and MGCC is 11.

### 4.2. Experimental Results Investigation

To evaluate the scenario of MG as an FR supporter, we define two cases to examine latency, reliabiliy, and security performance. In Case *I*, the MGCC power output follows the DSO requirements since the interaction is just on the DDS protocol side of our experimental setup. In Case II, the MGCC power output does not meet DSO requirements. Therefore, after using its optimization algorithm it issues commands to DER controller or BMS to justify their power output. Interaction in this case study is on both DDS and MMS protocol sides. Latency calculation for each case study is calculated as below:

(10) $${\mathrm{Latency}}_{CaseI}=T_{3}-T_{1}+T_{2}+T_{10}-T_{9}$$

(11) $${\mathrm{Latency}}_{CaseII}=\left(T_{5}\;or\;T_{4}\right)-T_{1}+T_{2}+T_{6}+T_{10}-\left(T_{7}\;or\;T_{8}\right)$$

where;

*T*_1_: time when DSO receives FR request from TSO;
*T*_2_: DSO optimization function time period;
*T*_3_: time when MGCC receives output power set point from DSO;
*T*_4_: time when DER receives output power set point from MGCC;
*T*_5_: time when BMS receives output power set point from MGCC;
*T*_6_: smart inverter ramp rate;
*T*_7_: time when the BMS send confirmation message to the MGCC;
*T*_8_: time when the DER send confirmation message to the MGCC;
*T*_9_: time when the MGCC send confirmation message to the DSO;
*T*_10_: time when the DSO send confirmation message to the TSO.

According to the mentioned experimental setup, we should consider delay time related to TSO interactions that are hidden in the DSO interactions. We also set $T_{6}$, smart inverter ramp rate, to zero since FR scenario needs immediate action [18]. Both case studies are tested for 1,000,000 times on the public Internet with different interval times between each test to examine the reliability of our communication testbed. The size of request messages is 850 bytes and 514 bytes for response messages. As discussed before, reliability is necessary for the request and response messages; therefore, to compare the performance of the system, it is set to both BEST_EFFORT and RELIABLE for each participant as QoS. In each packet loss situation, there is no attention to ensure the message gets to the destination in BEST_EFFORT mode. On the contrary, in RELIABLE state, retransmission of the data happens. The deadline of QoS for assuring messages are delivered to the DSO and MGCC is about 1000 ms. Since the Internet facilitates WAN communication, DDS security mode for each type of QoS is also applied to provide a secure infrastructure and comprehensive performance comparison.

**Figure 5:**
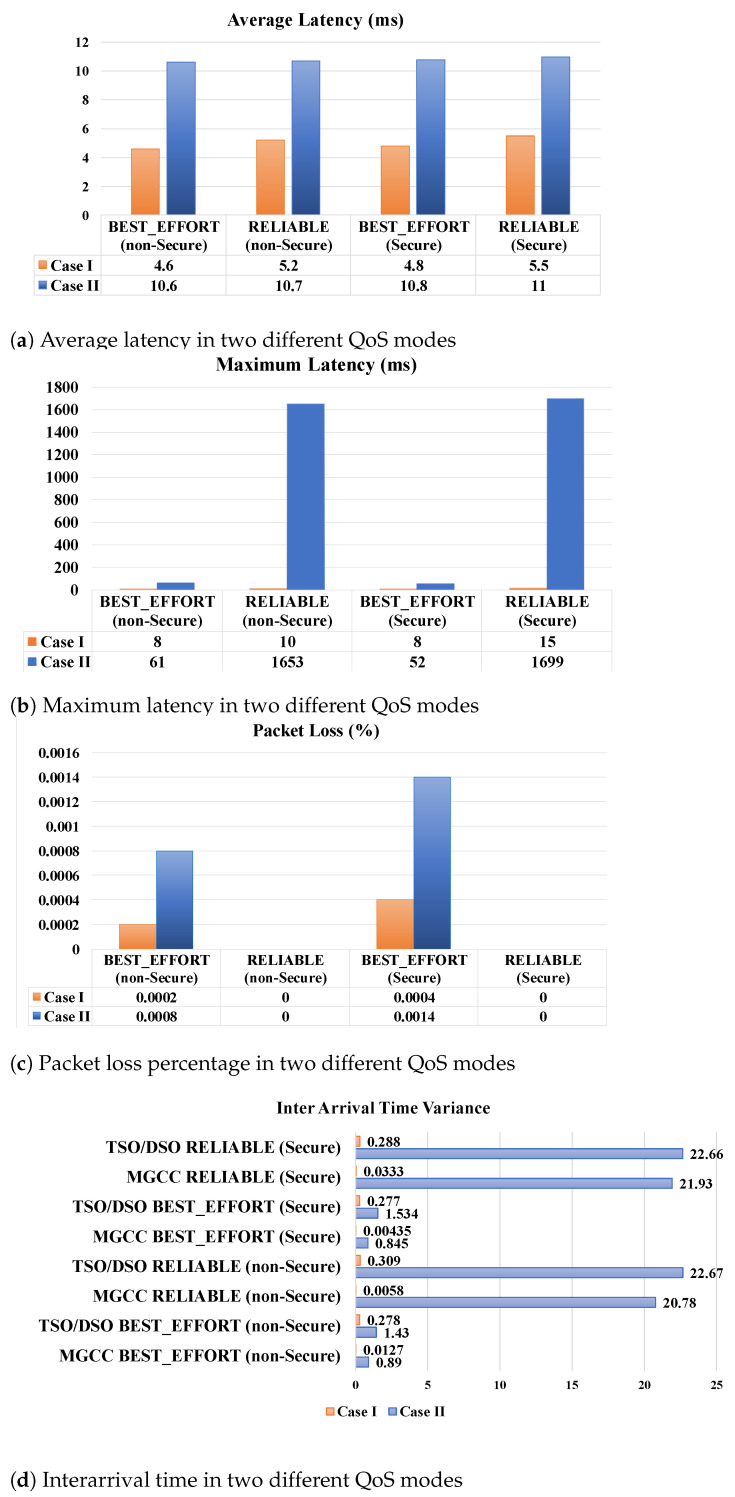
MG as FR-support case studies performance. The simulation results are demonstrated according to the QoS’s communication specification of the MG as the utility grid FR provision utilization, including latency, reliability, and security.

Figure 5 depicts the average and maximum latency, interarrival time variance, and message loss rate of test results. Overall, this figure shows that in RELIABLE and secure communication, there is the cost of rising latency. As expected, in Case *I*, pure DDS system, latency is less than Case II where the MGCC needs to negotiate with its elements on the MMS protocol. Looking at the detail, Figure 5a shows in the BEST_EFFORT mode, the average latencies of Case *I* are 4.6 and 4.8 ms for nonsecure and secure mode, respectively, which increased to 5.2 and 5.5 ms in RELIABLE mode. This increment in latency is due to the retransmission in any case of packet loss in RELIABLE mode from. Average latency in Case II doubled to 10.6 and 10.7 ms in nonsecure mode for BEST_EFFORT and RELIABLE modes respectively and these values change to 10.8 and 11 ms for secure mode. However, Figure 5c clarifies the importance of setting the RELIABLE mode of QoS for DDS as it has the effect of eliminating packet loss of the system.

Latency is not the only characteristic that is altered by reliable communication. Figure 5d reveals that interarrival time has also been affected by latency. Additionally noted, the variance of interarrival time in DSO is higher than MGCC. For example, in Case II, while the variance of interarrival time of MGCC for BEST_EFFORT and RELIABLE modes are 0.845 and 21.93 ms in secure mode, these variables for DSO, racketed to 1.534 and 22.66 ms. Due to DSO being on the client side, it should wait to receive a response from the server-side; however, any loss in request message will result in an increasing loss rate of DSO. If we look at the performance of the system in secure and nonsecure modes, the security provision of interaction will not cause the failure of the system achievement. Even in the strict situation, i.e., in Case II with Secure and RELIABLE mode, as shown in Figure 5b, the maximum latency is 1699 ms, which still abides by the time constraint requirement of FR.

In general, this experiment proves DDS can fulfill the time constraint requirements of the FR support scenario. However, we should not ignore the portion of processing time for optimization algorithms of both MGCC and DSO sides in the scenario success. Although the excellent latency performance of the pure DDS-based system in Case *I* validates the importance of scheduling MG performance to participate in AS provision of the utility grid, our results confirm the feasibility of MG deployment as an active element for the power grid frequency regulation.

## 5. Conclusions

From the introduction of smart inverters, the RESs, which are threads of power system stability due to their intermittence characteristics have a significant potential of turning into stability assistants. This unique plan can be viable through the supervision of MG equipped with ESS and RL when MG interacting with utility grid as an active element. Reliable control methodologies and especially communication infrastructure help this interaction happen. In this paper, we provided a scenario for MG as an FR support of utility grid. Regarding interoperability, the IEC 61850 information model and services applied along with the deployment of the DDS protocol for communication on the Internet to avoid employing dedicated infrastructure. Different situations of MG in response to main grid FR requirements implemented by deploying use cases of part 90–7 of IEC 61850.

As proof of our scenario, we provided an experimental setup and investigated time constraint representation in practice. The numerical results indicate DDS performance could meet the time constraints of FR. Although the excellent latency performance of the pure DDS-based system validates the importance of scheduling MG performance to participate in AS provision of the utility grid, our results confirm the feasibility of MG deployment as an active element of the power grid, which assists in contingency situations. As we implemented WAN on the Internet, RELIABLE applied as the QoS index in security mode. Although RELIABLE mode costs in longer delay, this parameter still stayed in the time boundaries that we require. 

## Figures and Tables

**Table 2:** LFC time constraint according to ENTSO regulations.

Control Level	$T_{p}$	$T_{d}$
Primary Control	1–2 s	15–30 s
Secondary Control	1–5 s	5–15 min
Tertiary Control	1 min	15 min

**Table 3:** Logical nodes and service requirements of micro-grid as FR-aid.

Step	Interaction		IEC 61850–90–7 Function	Parameters	LN					Service
	Sender	Receiver			Name	D.O.	CDC	FC	D.A.	
1	DER Controller	MGCC	DS93	Active Power Set point	DRCT	MaxWLim	ASG	SP	setMag	Report
				Active Power Output	MMXU	TotW	MV	MX	mag	
				Status	ZINV(PV)	GridModSt	ENS	ST	stVal,q,t	
					ZINV(ESS)	GridModSt	ENS	ST	stVal,q,t	
					DRCS	CHaSt	ENS	ST	stVal,q,t	
						VAPct	MV	MX	mag	
						VAChaPct			q t	
				Time	DPST	OpTms	INS	ST	stVal,q,t	
	MGCC	DSO	DS93	Nominal, Min and Max of Active Power	DOPR	ECPNomWRtg	ASG	SP	setMag	
								CF	minVal	
									maxVal	
				Status	DPST	ECPConn	SPS	ST	stVal,q,t	
					CSWI	POS	DPC	ST	stVal,q,t	
					MMXU	TotW	MV	MX	mag,q,t		
3	DSO	MGCC	INV1	Connect/Disconnect	CSWI	POS	DPC	ST	ctlVal	Operate
									stVal,q,t	Report
				Duration of Service Requirements	DOPM	RvrTms	ING	SP	setVal	SetDataValue-Request&Response
						WinTms				
						RmpTms				
			INV2	Active Power Set point	DRCT	WMaxLimPct	ASG	SP	setMag	
				Duration of Service Requirements	DOPM	RvrTms	ING	SP	setVal	
						WinTms				
						RmpTms				
3.1, 3.2, 3.3	MGCC	DER Controller	INV1	Connect/Disconnect	CSWI	POS	DPC	ST	ctlVal	Operate
									stVal,q,t	Report
				Duration of Service Requirements	DOPM	RvrTms	ING	SP	setVal	SetDataValue-Request&Response
						WinTms				
						RmpTms				
			INV2	Active Power Setpoint	DRCT	WMaxLimPct	ASG	SP	setMag	
				Duration of Service Requirements	DOPM	RvrTms	ING	SP	setVal	
						WinTms				
						RmpTms				
			INV4	Duration of Service Requirements	DOPM	RvrTms	ING	SP	setVal	
						WinTms				
						RmpTms				
				Set Charge/Discharge Rate	DRCT	OutWRte	ASG	SP	setMag	
					DOPM	OpModExlm	SPC	ST	ctlVal	Select
									stVal,q,t	Report

**Table 4:** Specification of experimental network infrastructure for MG as FR-support according to IEC 61850 standard.

Role in FR Scenario	Host Equipment	Hardware Specification			Software Specification	
		RAM	SSD	Processor	OS	Gateway Protocol
DSO	PC	32 GB	256 GB	Intel(R) Xeon(R) CPU @ 2.60 GHz	Ubuntu 16.04.6 LTS	DDS Client: 1OpenDDS-3.13
MGCC	Laptop	7.8 GB	256 GB	Intel (R) Core (TM) i7 CPU @ 2.50 GHz	Ubuntu 16.04.6 LTS	DDS Server:OpenDDS-3.13 IEC 61850 Client:mmslite V6.3
DER Controller	Raspberry-pi3	1 GB	microSDXC 64 GB	Broadcom BCM2837B0, Cortex-A53 64-bit SoC @ 1.4 GHz	Raspbian GNU/ Linux10	IEC 61850 Server:mmslite V6.3

## Nomenclatures

The following Nomenclatures are used in this manuscript:

α	Participation factor of ESS in FR
β	Participation factor of SG in FR
γ	Participation factor of LRES in FR
ξ	Participation factor of RL in FR
θ	Participation factor of MG in FR
*i*	Number of actors in the FR
D	Power system damping coefficient
H	System inertia provided by synchronous generators
$P_{d}$	Load power demand
$P_{ESS}$	Power delivered or consumed by ESS in the MG domain
$P_{LESS}$	Power delivered or consumed by large scale ESS
$P_{LRES}$	Power delivered by large scale RES
$P_{MG}$	Power delivered or consumed by the MG
$P_{mSG}$	Power delivered by micro-SG in the MG domain
$P_{RES}$	Power delivered by RES in the MG domain
$P_{RL}$	Power demanded by RL in the MG domain
$P_{SG}$	Power generated by SG

## Abbreviations

The following abbreviations are used in this manuscript:

ACL	Agent Communication Language
ADN	Active Distribution Network
AGC	Automatic Generation Control
AS	Ancillary Service
BMS	Battery Management System
DDS	Data Distribution Services
DER	Distributed Energy Resources
DSO	Distribution System Operator
EMS	Energy Management System
ENTSO	European Network of Transmission System Operators
ESS	Energy Storage Systems
FR	Frequency Regulation
GOOSE	Generic Object Oriented Substation Event
IED	Intelligent Electronic Devices
KT	Korean Telecommunication
LAN	Local Area Network
LESS	Large Scale Energy Storage Systems
LFC	Load Frequency Control
LRES	Large Scale Renewable Energy Sources
MAS	Multi-Agent System
MG	Microgrid
MGCC	Microgrid central controller
micro-SG	micro-Synchronous Generators
NGN	Next-Generation Network
OMG	Object Management Group
QoS	Quality of Services
RES	Renewable Energy Sources
RL	Responsive Loads
SG	Synchronous Generators
SISCO	Systems Integration Specialists Company
SMV	Sampled Measured Values Messages
TSO	Transmission System Operator
WAN	Wide-Area Network
XML	eXtensible Markup Language
